# TAK-242, a toll-like receptor 4 antagonist, against brain injury by alleviates autophagy and inflammation in rats

**DOI:** 10.1515/biol-2022-0662

**Published:** 2023-07-29

**Authors:** Yan Feng, Yaru Ju, Qiang Wu, Guozhu Sun, Zhongjie Yan

**Affiliations:** Department of Neurosurgery, The Second Hospital of Hebei Medical University, Shi Jiazhuang, Hebei 050000, China; Perinatal Center, The Fourth Hospital of Shi Jiazhuang, Shi Jiazhuang, Hebei 050011, China; Department of Neurosurgery, The Second Hospital of Hebei Medical University, No. 215 Heping West Road, Shi Jiazhuang, Hebei 050000, China

**Keywords:** toll-like receptor 4, TAK-242, autophagy, neuroinflammation, traumatic brain injury

## Abstract

Inhibition of Toll-like receptor 4 (TLR4)-mediated inflammatory pathways exerts a critical effect on neuronal death; therefore, it is a possible new therapeutic approach for traumatic brain injury (TBI). Resatorvid (TAK-242) is a novel small-molecule compound widely used to inhibit TLR4-mediated pathways, but the protective mechanism of TAK-242 in TBI remains unclear. Herein, we analyzed the neuroprotective effects of TAK-242 in rats after TBI. The rat model of brain injury was established using a modified Free-fall device, and the rats were injected with TAK-242 (0.5 mg/kg) through the caudal vein before TBI. The rats were allocated into four groups: a sham group, a TBI group, a TBI + vehicle group, and a TBI + TAK-242 group. The brain tissue was extracted for histology and determination of the expression of autophagy-related proteins and inflammatory mediators. TAK-242 pretreatment significantly reduced the damage to hippocampal neurons. Neuronal autophagy increased after brain injury, whereas TAK-242 significantly reduced autophagy marker protein LC3-II in the hippocampus. In addition, TAK-242 pretreatment significantly downregulated NF-κB p65, TNF-α, and IL-1β in the hippocampus. In conclusion, TAK-242 significantly reduced hippocampal neuronal damage by inhibiting autophagy and neuroinflammatory activity, possibly via the NF-κB signaling pathway.

## Graphical abstract

**Figure:**
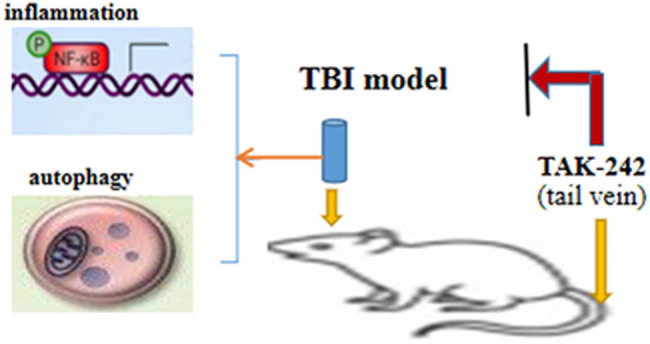

## 1 Introduction

As a significant contributor to the mortality of young people worldwide, traumatic brain injury (TBI) often places a heavy financial burden on families/society. Based on pathology, TBI is classified as a primary brain injury due to the immediate destruction of brain tissue and a secondary brain injury because of cellular and molecular events after injury [1]. These events promote mitochondrial dysfunction/biochemical cell-death signaling, ultimately leading to neuronal cell damage together with functional deficits [2,3]. Studies in the last decade have proved that innate immunity and inflammatory responses are associated with neurological deficits based on the release of endogenous ligands such as Toll-like receptors (TLRs) [4,5]. Among these TLRs, a pattern recognition receptor for innate immune responders, TLR4, is widespread in the brain and induces an inflammatory response after ischemic stroke or brain injury [6,7]. Myeloid differentiation factor 88 (MyD88) is an endogenous adaptor protein that activates downstream NF-κB by TLR4 agonists as well as families of IL-1 receptors, playing a critical role in coordinating the production of proinflammatory cytokines [8,9].

In stressed cells, autophagy is related to the degradation of proteins and entire organelles [10]. Our preliminary experiments demonstrated that TBI not only activated autophagy but also increased the expression of microtubule-associated protein 1 light chain 3 in neurons, and suppression of neuronal autophagy was neuroprotective in the rat hippocampus [11,12]. Autophagy has been increasingly acknowledged as a crucial factor in an innate immune response, which plays an important role in immunity and inflammation, as well as metabolism and cell survival. In addition, TLR4 acts as an environmental detector of autophagy [13,14]. The selective autophagy of aggresome-like-induced structures has been demonstrated in response to TLR4 stimulation in macrophages [15], and TLR4-dependent autophagy is critical for macrophage-associated inflammatory responses [16]. However, whether TLR4 is associated with neuroinflammation and the autophagic death of neuronal cells after TBI remains unclear.

As a small-molecule TLR4 inhibitor, resatorvid (TAK-242) selectively inhibits TLR4 signaling by binding to Cys747 within the cells [17]. It is safe in humans and is currently under clinical development as a possible therapeutic agent for the treatment of sepsis [18]. TAK-242 also minimizes target organ damage and systemic inflammation in animal models [19]. Importantly, TAK-242 may penetrate the blood–brain barrier to be rapidly distributed and provide neuroprotective effects in a variety of other diseases associated with cerebral hemorrhage and inflammation [20]. Pretreatment with TAK-242 yielded beneficial protection from gram-negative sepsis/peritonitis induced by lipopolysaccharide. TLR4 may be a novel intervention for intracerebral hemorrhage and TBI [21,22]. Therefore, this study was designed to explore the role of TAK-242 in hippocampal neuronal damage using a rat model, showing that TAK-242 might have potential as a novel target for TBI, achieving protection through suppressing autophagy and neuroinflammation.

In this study, we examined the role of TAK-242 in hippocampal neuronal damage using H&E staining in a rat model. Neuronal autophagy and inflammatory cytokines expression were also assessed. Results proved that TAK-242 might have a potential as a novel target for TBI, achieving protection through suppressing autophagy and neuroinflammation.

## 2 Materials and methods

### 2.1 Animals and TBI model

The experiments were performed as per the guidelines of the Chinese Council on Animal Protection and ratified by the Animal Ethics Committee of Hebei Medical University. In total, 100 SD rats (male, 280–330 g, Shijiazhuang, China) were kept in a pathogen-free room under a normal 12-h cycle of light and dark and had *ad libitum* access to food and water before/after surgery. The rat TBI model was established with the application of a modified Free-fall device [23]. Briefly, after anesthesia using isoflurane (4% induction and 2% maintenance), a longitudinal incision along the midline was made to expose the skull before a steel disk (10 mm in diameter and 3 mm in thickness) was fixed to the skull using dental acrylic. Subsequently, rats were positioned on top of a foam mattress, and a 40 g weight was dropped vertically from a height of 1.5 m directly onto the disk to cause a diffuse brain injury. The sham group was subjected to the same surgical procedure but without TBI. To guarantee normal body temperature within the recovery period, the rats were kept on 37°C heat pads for 24 h. The model generally caused moderate brain damage in rats, and there was little difference in the severity of the damage during the model preparation. The extent of brain damage was measured by modified neurological severity scores [24], as shown in Table 1. A total of 100 rats were used in this study, and three died of brain damage after TBI.

**Table 1:** Comparison of the score of nerve damage in each group ( \bar{\chi }] ± *s*)

Group	Sham	TBI	TBI + vehicle	TBI + TAK-242
24 h after TBI (score)	0	9 ± 2.8	9 ± 3.6	8 ± 3.4


**Ethical approval:** The research related to animal use has complied with all the relevant national regulations and institutional policies for the care and use of animals and has been approved by the Animal Ethics Committee of Hebei Medical University Animal Center (approval ID: 202203146).

### 2.2 Experimental groups and drug administration

A random number table was used to assign the rats to four groups (*n* = 5): a sham group, a TBI group, a TBI + vehicle group, and a TBI + Resatorvid (TAK-242) group. The rats were sacrificed at 6, 12, 24, 48, or 72 h after TBI. TAK-242 (Millipore) was dissolved in 1% dimethyl sulfoxide (DMSO) and 0.9% saline at a concentration of 0.4 mg/ml before being intravenously (i.v.) injected (0.5 mg/kg, approximately 10 s) into the tail vein 10 min before TBI induction, as described previously [25]. The sham/TBI group was i.v. injected with an equal amount of saline, whereas the vehicle group was injected with equal volumes of DMSO/saline.

### 2.3 Histological analysis

The rats were anesthetized 24 h after TBI, and the heart was perfused with 4% paraformaldehyde (PFA). The harvested brains were fixed in 4% PFA and then subjected to gradient ethanol dehydration, xylene transparency, and paraffin embedding following the standard histological procedure. The brain was serially sectioned (5 μm thick sections), and the sections (approximately 1.9 mm) in the CA1 area posterior to the bregma were analyzed, with the density of pyramidal cells (cells/mm) counted following H&E staining. Cell counts were performed under an optical microscope for active neurons with well-ordered cells, abundant cytoplasm, and clear nuclei.

### 2.4 Immunofluorescence analysis

The fixed brain tissue was immersed in sucrose solution (30%) and then embedded in optimal cutting temperature compound before serial sections (15 μm thick) of the hippocampus were cut using a frozen slicer (bregma 1.90–3.00) and Triton X-100 treatment (0.4%, 30 min). Subsequently, the sections were blocked with normal donkey serum (1 h) before incubation overnight at 4°C with the primary rabbit anti-LC3 polyclonal antibody (1:100; Medical & Biological Laboratories Co. (MBL)) and mouse anti-NeuN monoclonal antibody (1:100; Millipore). After phosphate-buffered saline washing, the sections were incubated for 2 h at 37°C with the corresponding Alexa Fluor® 594 (anti-rabbit IgG) or Alexa Fluor® 488 (anti-mouse IgG) conjugated antibodies (1:1,000; Santa Cruz Biotechnology) in the dark. Finally, the sections were counterstained with 4′,6-diamidino-2-phenylindole (DAPI), followed by sealing with an anti-quenching agent and viewed with a laser scanning confocal microscope (Olympus Fluoview™ FV1000).

### 2.5 Western blotting

After the rapid isolation of the hippocampus, the total protein was extracted and then quantified using the enhanced bicinchoninic acid protein assay kit (Solarbio, Beijing, China). The protein samples (50 μg) were separated by sodium dodecylsulfate-polyacrylamide gel electrophoresis and transferred to polyvinylidene fluoride membranes using transfer apparatus (200 mA, 50 min). The membranes were blocked using skimmed milk powder (5%) (2 h, room temperature), followed by overnight incubation at 4°C with primary rabbit anti-LC3 (1:1,000; MBL), rabbit anti-NF-κB p65, rabbit anti-TNF-α (1:500; Affinity), rabbit anti-IL-1β (1:500; Affinity), and rabbit anti-β-actin (1:1,000; Affinity). Then, the membranes were incubated with the appropriate peroxidase-conjugated secondary antibodies (1:5,000 anti-rabbit IgG; Santa Cruz Biotechnology) for 2 h at room temperature before being developed and fixed with enhanced chemiluminescence in a dark room. The bands were visualized using Image Lab 4.1 (Bio-Rad) and quantified using ImageJ software with β-actin as a loading control.

### 2.6 Statistical analysis

The data were presented as mean ± standard deviation (SD). SPSS 19.0 software (IBM, Armonk, NY, USA) was used for data analysis. Data comparison among multiple groups was conducted with one-way analysis of variance, and statistical analysis between two groups was performed with the Student-Newman–Keuls post hoc tests. Sample sizes subjected to statistical analysis were at least 5 animals per group (*n* = 5). Differences were considered significant at *p* < 0.05.

## 3 Results

### 3.1 TAK-242 treatment alleviated hippocampal neuron damage

**Figure 1:**
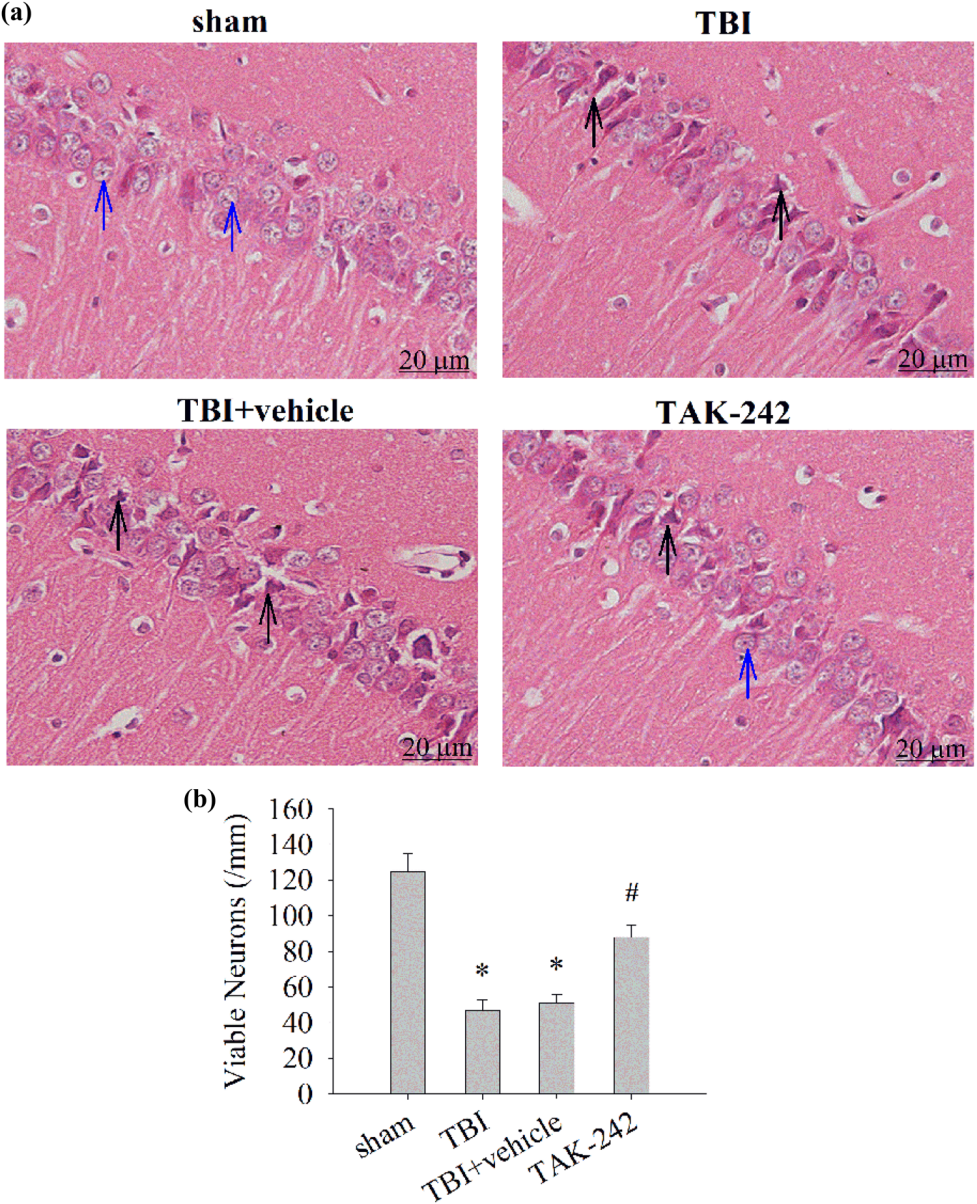
The effect of TAK-242 on hippocampal neuronal damage by H&E staining. (a) Representative staining in the hippocampal CA1 area after sham, TBI, TBI + vehicle, and TAK-242 groups at 24 h (magnification, 20 μm). Normal hippocampal neurons in sham group (blue arrow), nucleolus shrinkage, neuronal degeneration, and necrosis in TBI or TBI + vehicle group (black arrow). (b) Quantification of viable neurons per millimeter of CA1 in each group. (*n* = 5, per group; *p < 0.01 vs sham group; ^#^ *p* < 0.05 vs TBI group).

H&E staining was performed to examine the effect of TAK-242 on hippocampal neuronal damage 24 h after TBI. In contrast to sham controls, morphological alterations were observed in CA1 pyramidal neurons accompanied by neuronal loss in TBI or TBI + vehicle animals (Figure 1, *p < 0.01 vs sham group). TAK-242 treatment not only moderated the morphologic alterations but also decreased the neuronal loss related to TBI (^#^ *p* < 0.05 vs TBI group).

### 3.2 TAK-242 treatment inhibited autophagy in hippocampal neurons

**Figure 2:**
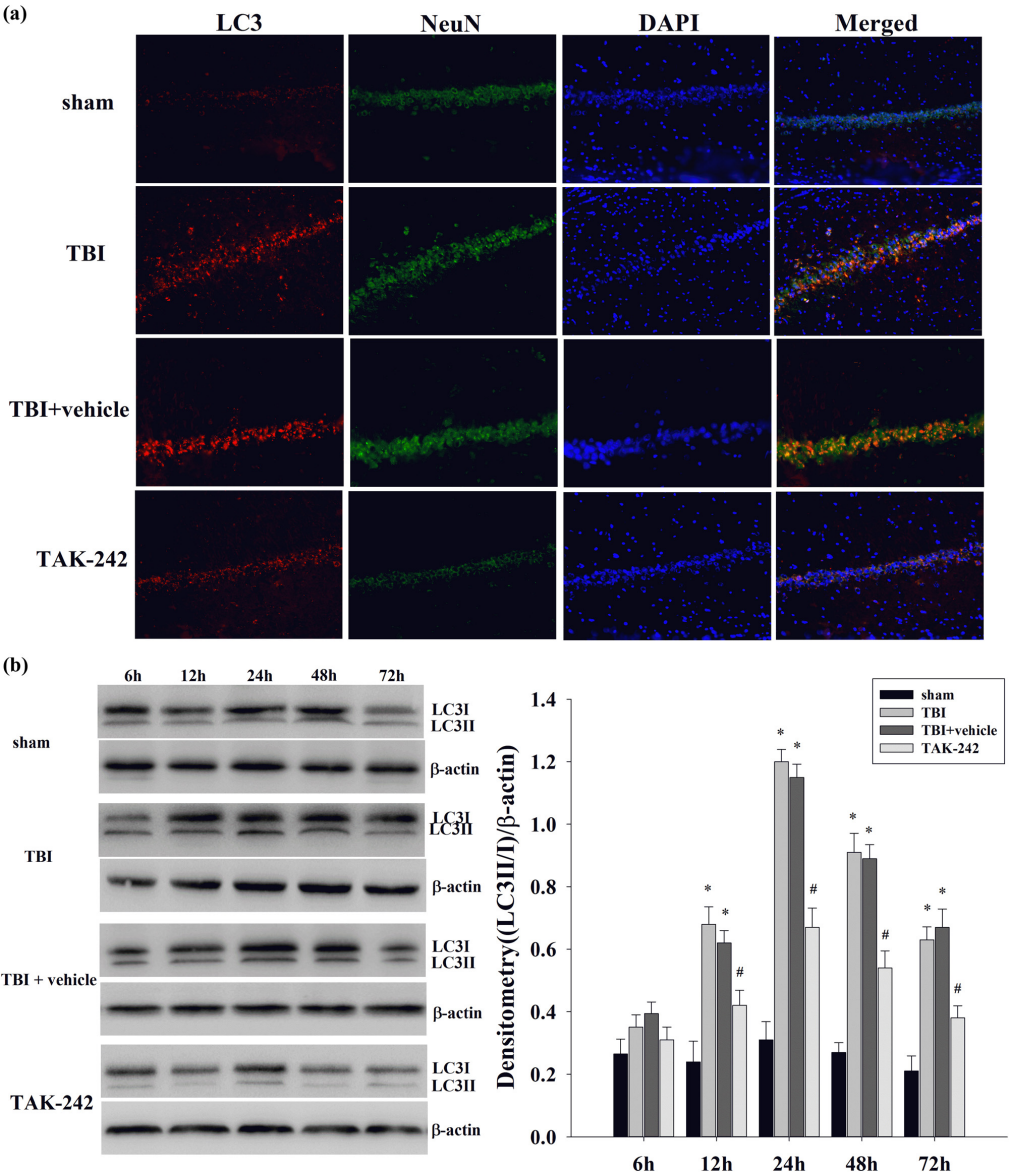
The effect of TAK-242 on neuronal autophagy. (a) Co-localization of LC3 and NeuN at 24 h post-TBI was determined by immunofluorescent staining (magnification, 50 μm), and cell nuclei were counterstained by DAPI. The orange coloring in the merged image suggests co-localization. (b) Western blot shows the levels of LC3 and β-actin in hippocampus at 6, 12, 24, 48, and 72 h in TBI or sham surgery. Densitometry analysis of LC3-II/I band was corresponding to β-actin. LC3-II/I was significantly elevated from 12 to 72 h in TBI or TBI + vehicle group (*p < 0.01 vs sham group). TAK-242 treatment significantly decreased the level of LC3-II/I protein expression (^#^ *p* < 0.05 vs TBI group). Data were expressed as mean ± SD (*n* = 5 per group).

To assess TAK-242’s effect on autophagic activity in brain damage post-TBI, immunofluorescence staining was doubly carried out to analyze the co-localization of LC3 and NeuN expression. The co-localization of LC3 (red) and NeuN (green) expression in hippocampal neurons is shown in Figure 2a with the DAPI-stained nuclei (blue). LC3 immunoreactivity (red) was observed in NeuN-positive cells (green) 24 h post-TBI, confirming that most TBI-induced autophagy occurred in the neurons. Subsequently, we tested if TAK-242 treatment suppressed LC3 expression from 6 to 72 h post-TBI. Figure 2b reveals that in the hippocampus, LC3-II/I protein levels significantly increased at 12 h in the TBI or TBI + vehicle groups and remained high until 72 h post-injury (*p < 0.01 vs sham group). In addition, TAK-242 pretreatment significantly decreased LC3-II/I expression (^#^ *p* < 0.05 vs TBI group).

### 3.3 TAK-242 treatment decreases NF-κB expression in the hippocampus

**Figure 3:**
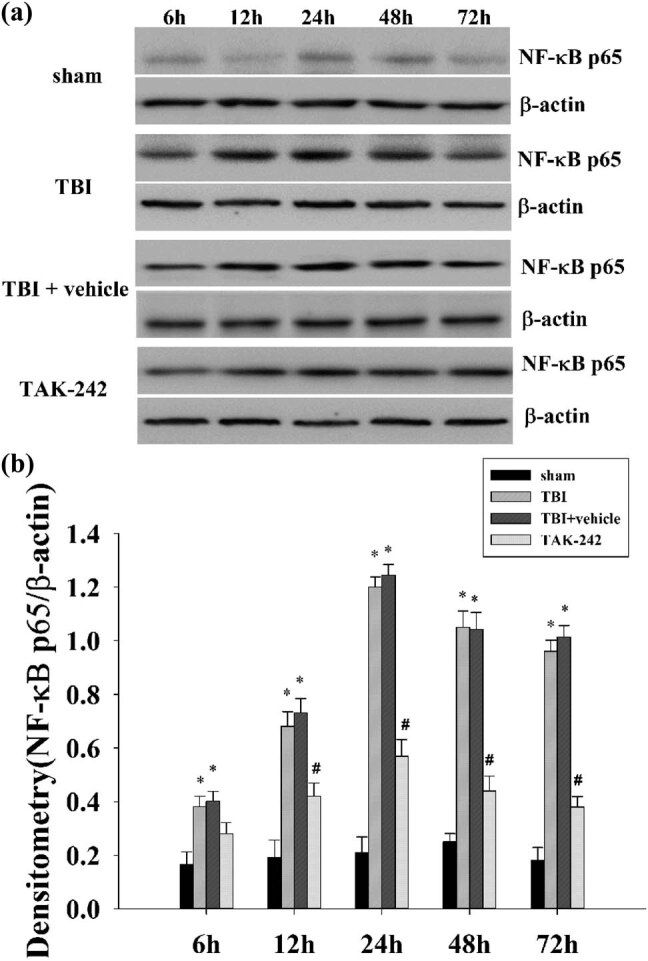
(a) Western blot analysis demonstrates levels of NF-κB p65 in the hippocampus of rats from 6 to 72 h in sham, TBI, TBI + vehicle, or TAK-242 group (*n* = 5 per group). (b) Densitometry of the NF-κB p65 band correlates to the β-actin band. Results demonstrated TBI-induced NF-κB p65 activation (*p < 0.01 vs sham group); and TAK-242 treatment significantly reduced the level of NF-κB p65 protein expression at 12, 24, 48, and 72 h post-TBI (^#^ *p* < 0.05 vs TBI group). The bars represent mean ± SD (*n* = 5 per group).

To further explore the role of TAK-242 in neuroinflammation post-TBI, the expression of NF-κB p65, a signaling molecule downstream of TLR4, was assessed at 6, 12, 24, 48, and 72 h post-TBI. As demonstrated in Figure 3, compared to the sham group, there was significant upregulation of NF-κB p65 expression in the TBI/TBI + vehicle group (*p < 0.01 vs sham group). TAK-242 treatment attenuated NF-κB p65 expression in rats’ hippocampus from 12 to 72 h in contrast to the TBI group with statistical significance, among which the protein level peaked at 24 h (^#^ *p* < 0.05 vs TBI group).

### 3.4 TAK-242 treatment decreased the levels of TNF-α/IL-1β in the hippocampus

**Figure 4:**
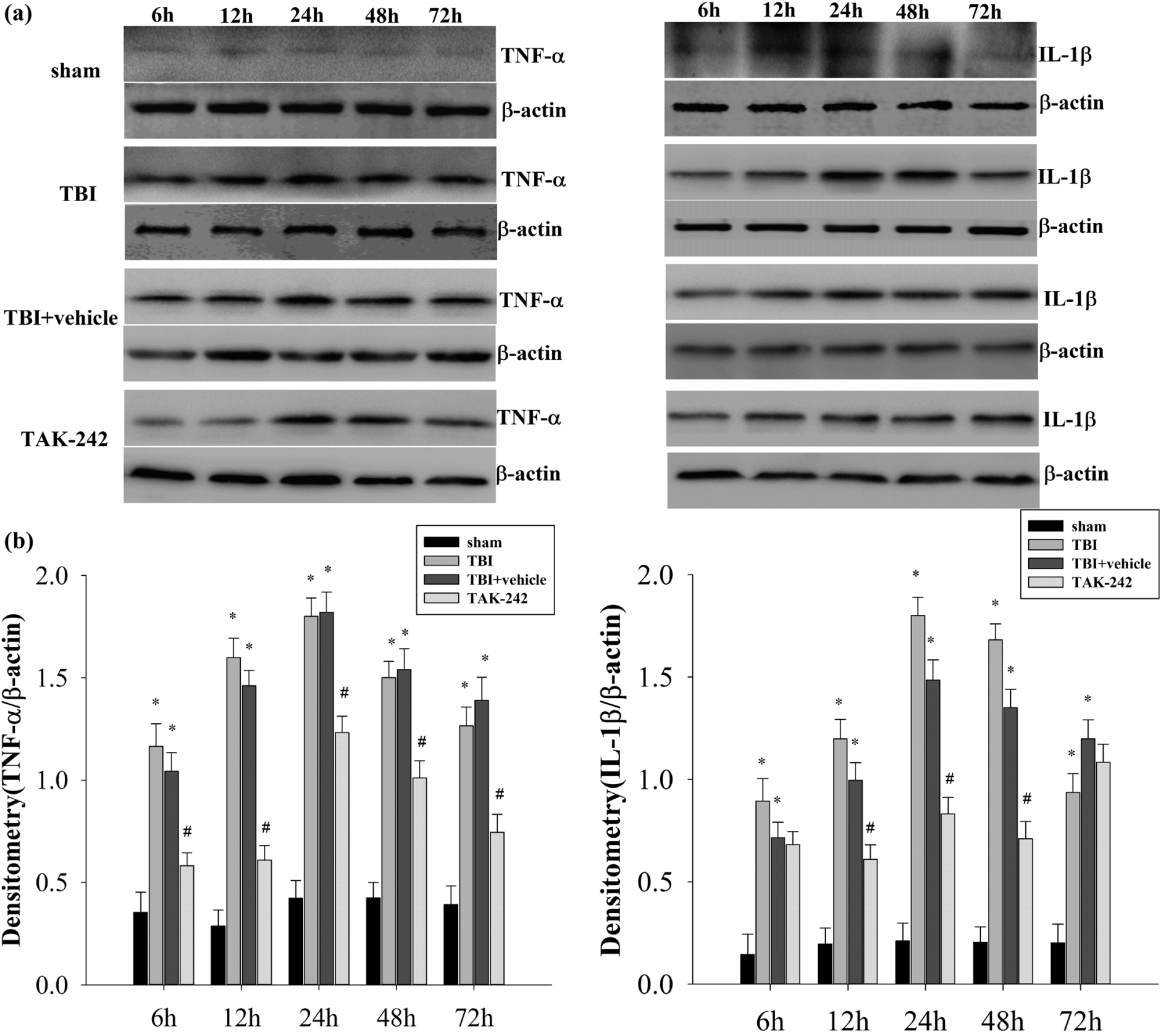
(a) Western blot analysis demonstrated levels of TNF-α and IL-1β in the hippocampus of rats from 6 to 72 h in sham, TBI, TBI + vehicle, or TAK-242 group (*n* = 5 per group). (b) Densitometry of the TNF-α and IL-1β band correlates to the β-actin band. Results demonstrated a significant increase in TNF-α and IL-1β expressions in TBI or TBI + vehicle group (*p < 0.01 vs sham group); and TAK-242 treatment significantly downregulated the levels of TNF-α and IL-1β at 12, 24, and 48 h post-TBI (^#^ *p* < 0.05 vs TBI group). The bars represent mean ± SD (*n* = 5 per group).

Elevated levels of proinflammatory cytokines have been demonstrated to be positively correlated with the severity of TBI after brain damage [26]. Therefore, we evaluated TNF-α/IL-1β levels, showing that they significantly increased in the TBI/TBI + vehicle group in contrast to the sham group at 6, 12, 24, 48, and 72 h with the greatest increase at 24 h post-TBI (Figure 4, *p < 0.01 vs sham group). The upregulation of TNF-α and IL-1β levels was significantly downregulated by TAK-242 pretreatment (^#^ *p* < 0.05 vs TBI group). Taken together, these results suggest that TAK-242 might have the potential to reduce TNF-α and IL-1β levels in the hippocampus of a TBI model.

## 4 Discussion

Based on mechanisms related to primary and secondary brain injuries, TBI is considered a highly complicated disorder. Extensive preclinical animal studies have been conducted to develop treatments for TBI. However, to date, no pharmacological treatment has been translated into common clinical practice; therefore, novel strategies for TBI are imperative. Preclinical data have demonstrated that TLR4 exerts a critical impact on neuronal death and thus may be a potential effective intervention for TBI [21,27]. Meanwhile, TAK-242, a small-molecule compound that selectively inhibits TLR4 signaling, is neuroprotective in cerebral ischemia or intracerebral hemorrhage [22,28]. Herein, we investigated the neuroprotective effect of TAK-242 in rats with TBI, showing that TAK-242 could reduce hippocampal neuron damage by inhibiting autophagy and neuroinflammatory activity, and the mechanism may be related to the NF-κB signaling pathway.

Male SD rats were used in this study because the hormonal changes caused by the physiological cycle of female animals are relatively large, which may affect the experimental results. It has also been reported that estrogen has a protective effect against brain damage [29,30], so estrogen interference in female rats may affect the model or the results of the drug administration. A sex bias in TLR activation has already been shown, with women exhibiting increased cellular- and humoral-mediated immune responses and a higher risk of autoimmune disease compared to men [31]. Sex-related differences in immune activity have been well documented and are known to extend to the brain [32]. Doyle et al. reported that TLR4 inhibition enhanced the analgesic effects of morphine in female rats but not in male rats [33]. Male and female TLR4^−/−^ mice also show region-specific changes in brain-derived neurotrophic factor (BDNF) expression, as TLR4^−/−^ females show reduced hippocampal BDNF expression compared to wild type (WT) females, whereas no differences were seen between the male TLR4^−/−^ and WT mice [34]. However, there is still a lack of scientific and experimental knowledge about sex differences in TLR function and traumatic craniocerebral injury, so future studies will use female mice or TLR4^−/−^ mice to further investigate the neuroprotective effect of TAK-242 after brain injury.

Our previous research has shown that TAK-242 may have a neuroprotective effect by inhibiting neuronal autophagy and the TLR4-mediated inflammatory pathway in a TBI rat model [11]. However, this study has some shortcomings and is a complement and extension of the previous study. First, we did not clearly describe the morphological changes in rat brain tissue after injury. In this study, TAK-242 was protective against trauma-induced hippocampus neuron damage in the rat TBI model. In contrast to the TBI group, TAK-242 pretreatment not only moderated the morphologic alterations but also decreased the neuronal loss related to TBI. Therefore, TAK-242 was protective against trauma-induced hippocampus neuron damage in the rat TBI model consistent with previous studies suggesting the neuroprotective potential of TAK-242 post-brain injury [11,21,27]. In addition, recent research confirms that DMSO is suitable for use in studies investigating neuroprotective treatment strategies as it does not influence post-traumatic brain damage [35]. However, Bulama et al. showed that DMSO improves the functional recovery of cognitive function in rats after TBI by increasing the level of antioxidant enzymes that quench and inhibit the formation of reactive oxygen species [36]. Therefore, to eliminate the influence of DMSO on the experimental results, we added the vehicle group. The results were consistent with the former with no statistical difference between the TBI and TBI + vehicle groups, indicating that DMSO did not affect our results after brain injury in rats.

Previous studies have also reported that TAK-242 alleviates brain edema and neurological deficits following TBI [11], and therefore, we did not conduct repeated verification in this study. Studies on the effects of TAK-242 on hypoxic-ischemic encephalopathy (HIE) showed that TAK-242 alleviates neurological deficits and improves neurobehavioral function in a neonatal HIE rat model [37]. In addition, TAK-242 was similarly confirmed to improve the neurobehavior of intraventricular hemorrhage rats and attenuate their learning and spatial memory deficits, suggesting early TAK-242 intervention is beneficial for blocking the progression of neurocognitive deficits induced by hydrocephalus [22].

It has been demonstrated that neuronal autophagy is induced at 6 h or earlier, peaking at 24 h and lasting until 72 h in the hippocampus following TBI [38–40]. Compared with our previous study, we added two additional time points, namely, 6 and 72 h, but did not observe autophagy occurring at 6 h after injury, and the expression of LC3-II/I protein in the hippocampus was significantly upregulated 12 h following TBI or in the TBI + vehicle group and persisted for 72 h following injury. This may be related to the severity of brain damage in rats, which is also a question for subsequent studies. Accumulating evidence suggests that autophagy is related to secondary brain injury after TBI, so if the pathway is inhibited, it may reduce the degree of brain injury and improve the deficits in functional outcomes [11,41,42]. However, Lipinski et al. [43] have shown that augmented and/or restored autophagy flux directly reduces neuronal cell death and neuroinflammation and promotes re-myelination necessary for long-term recovery, which is also a potential treatment strategy after TBI and spinal cord injury. In the present study, our results show that the enhancement of neuronal autophagy is consistent with the severity of neuronal injury after brain injury in rats. Neurons are terminally differentiated cells that last throughout the life cycle of the organism, so autophagy is particularly important in neurons [44,45]. As our results clarified alterations, most autophagy induced following TBI occurred in neurons. In addition, TAK-242 can remarkably reduce the occurrence of neuronal injury and autophagy. Therefore, it could be inferred that the neuroprotective impact of TAK-242 on brain injury may decrease neuronal autophagy.

The neuroprotective mechanism of TAK-242-associated alleviation of neuronal autophagy in the hippocampus is unclear. Herein, we hypothesized that the suppressed neuronal autophagy responds to anti-inflammatory properties via the down-regulation of NF-κB expression. Studies have shown that protein expression of TLR4 is upregulated after TBI and reaches a maximum at 24 hours. Compared with wild-type mice, TLR4^−/−^ mice showed attenuated functional impairment, brain edema and cytokine release post-TBI [46]. TLR4 can activate IκB phosphorylation/degradation to translocate NF-κB from the cytoplasm to the nucleus, further inducing the expression of inflammatory mediators [47]. NF-κB, an essential nuclear transcription factor, participates in immune/inflammatory responses. It could be activated when cells receive pathological stimuli, leading to nuclear translocation and consequently inducing the expression of TNF-α or IL-1β observed in secondary brain injury post-TBI [48]. The present study showed that there was a significant upregulation of NF-κB p65, TNF-α, and IL-1β expression from 6 to 72 h in the TBI group or TBI + vehicle group compared to the sham group. Also, TAK-242 pre-treatment significantly reduced the expression of NF-κB p65 and its downstream inflammatory factors in the rat hippocampus from 12 to 72 h. Dong et al. showed that TAK-242 pretreatment ameliorates epileptic symptoms in mice via inhibition of the TLR4/NF-κB inflammatory pathway [49]. Recent studies have shown that TAK-242 inhibits the activation of extracellular signal-regulated kinases and NF-κB pathways and further reduces Fra-1 expression during ischemia–reperfusion injury in rats [50]. So, it could be inferred that neuronal autophagy could be inhibited by TAK-242 pretreatment in the rat hippocampus, and the autophagic pathway could be affected by TAK-242 based on the downregulation of the TLR4/NF-κB signaling cascade.

In conclusion, TAK-242 pretreatment of a rat TBI model remarkably alleviated hippocampal neuron damage and thus is a potential treatment to inhibit neuronal autophagy and neuroinflammation via the TLR4/NF-κB signaling cascade, providing an insight into the underlying mechanism of TAK-242 in TBI.
